# The visual nonverbal memory trace is fragile when actively maintained, but endures passively for tens of seconds

**DOI:** 10.3758/s13421-019-01003-6

**Published:** 2019-12-23

**Authors:** Denis McKeown, Tom Mercer, Kinga Bugajska, Paul Duffy, Emma Barker

**Affiliations:** 1 School of Psychology, University of Leeds, Leeds, LS2 9JT UK; 2 Department of Psychology, University of Wolverhampton, Wolverhampton, WV1 1LY UK

**Keywords:** Visual memory, Proactive interference, Decay, Forgetting, Retro-cue

## Abstract

Despite attempts at *active* maintenance in the focus of attention, the fragile nature of the visual nonverbal memory trace may be revealed when the retention interval between target memoranda and probed recall on a trial is extended. In contrast, a *passive*ly maintained or unattended visual memory trace may be revealed as persisting proactive interference extending across quite extended intervals between trials in a recent probes task. The present study, comprising five experiments, used this task to explore the persistence of such a passive visual memory trace over time. Participants viewed some target visual items (for example, abstract colored patterns) followed by a variable retention interval and a probe item. The task was to report whether the probe matched one of the targets or not. A decaying active memory trace was indicated by poorer performance as the memory retention interval was extended on a trial. However, when the probe was a member of the target set from the *preceding* trial, task performance was poorer than a comparison novel probe, demonstrating proactive interference. Manipulations of the intertrial interval revealed that the temporal persistence of the passive memory trace of an old target was impressive, and proactive interference was largely resilient to a simple ‘cued forgetting’ manipulation. These data support the proposed two-process memory conception (active–passive memory) contrasting fragile active memory traces decaying over a few seconds with robust passive traces extending to tens of seconds.

Events from the immediate past can persist into our present and disrupt the formation of new memories. Researchers have long been aware of such ‘proactive interference’ (PI; e.g., Loess, 1964; Underwood, 1948; Whitely, 1927) and it remains the subject of extensive research interest (e.g., Devkar & Wright, 2016; Jonides & Nee, 2006; Makovski & Jiang, 2008). One way of observing PI is through the recent probes task (Atkinson, Hermann, & Wescourt, 1974; Monsell, 1978). Participants retain an array of targets over a brief delay and then determine whether a single probe matches one of the targets. On ‘positive’ trials there is a match, but mismatch trials can take two forms—'nonrecent’ probes (NRP) are either completely novel or not experienced for multiple trials, whereas ‘recent probes’ (RP) match a target from the *previous* trial. PI is manifested when responding to RP stimuli is slower and less accurate than responding to NRP stimuli, which we term the ‘recent probe effect’.

Importantly, the presence of PI can provide insights into the continued availability of old, residual memories, which has implications for theories of forgetting. For example, temporal decay theory expects old items to be gradually forgotten, so extending the intertrial interval (ITI) within the recent probes task should allow RP stimuli to decay and PI to vanish. Temporal distinctiveness models (e.g., Brown, Neath, & Chater, 2007) also predict a reduction in PI over time. In temporal distinctiveness accounts, memories are forgotten through PI, which is especially likely in crowded temporal contexts (e.g., when competing to-be-remembered items occur in close temporal proximity). Consequently, isolating items in time *should reduce PI*, and over a long ITI there should be less likelihood of confusing events on the current trial with those from the previous trial.

Berman, Jonides, and Lewis (2009) manipulated the ITI within the recent probes task using verbal memoranda, but, in contrast to time-based theories relying on decay or temporal distinctiveness, found time-insensitive PI. Yet PI effects may differ if nonverbal, visual stimuli are employed, as visual WM may show rapid time-dependent forgetting (e.g., Ricker & Cowan, 2010, 2014). As such, PI for unfamiliar visual material may be immediate and transient (e.g., Lin & Luck, 2012), and limited to very brief “carryover” effects (e.g., Makovski & Jiang, 2008). Over longer delays, it is reasonable to expect PI for visual stimuli to disappear, in line with a decay process (see Mercer & Duffy, 2015).

Contrary to these expectations, McKeown, Holt, Delvenne, Smith, and Griffiths (2014) observed long-lasting PI in a recent probes task using novel, abstract visual patterns. Their experiment manipulated both the within-trial retention interval (RI) and the between-trial ITI (each lasting 1 s or 6 s). Extending the RI led to slower and less accurate responding, but extending the ITI did not diminish PI. McKeown et al. offered a novel conception proposing a form of enduring memory representation which preserves fine details of recent stimuli, is resistant to decay over tens of seconds, and is able to withstand nonspecific interference. This form of storage they termed *passive* memory, to distinguish it from *active* memory traces that may be attentionally maintained, subject to temporal decay and vulnerable to interference by subsequent stimuli. The proposal was that the attended target items on the current trial of a recent-probes task decay, but the passive traces of prior trial items do not.

The time course of PI therefore has important theoretical implications for theories of forgetting (e.g., Barrouillet, Uittenhove, Lucidi, & Langerock, 2018; Ricker, Vergauwe, & Cowan, 2016); for the plausibility of decay in WM (e.g., McKeown, Mills & Mercer, 2011; McKeown & Mercer, 2012; Mercer, 2014; Mercer & McKeown, 2014; Rademaker, Park, Sack, & Tong, 2018; Schneegans & Bays, 2018); and for McKeown et al.’s (2014) active–passive dual memory process. The present study further explored the time course of PI and examined the role of active versus passive maintenance and top-down control over PI.

## Methodology and analysis

**Fig. 1:**
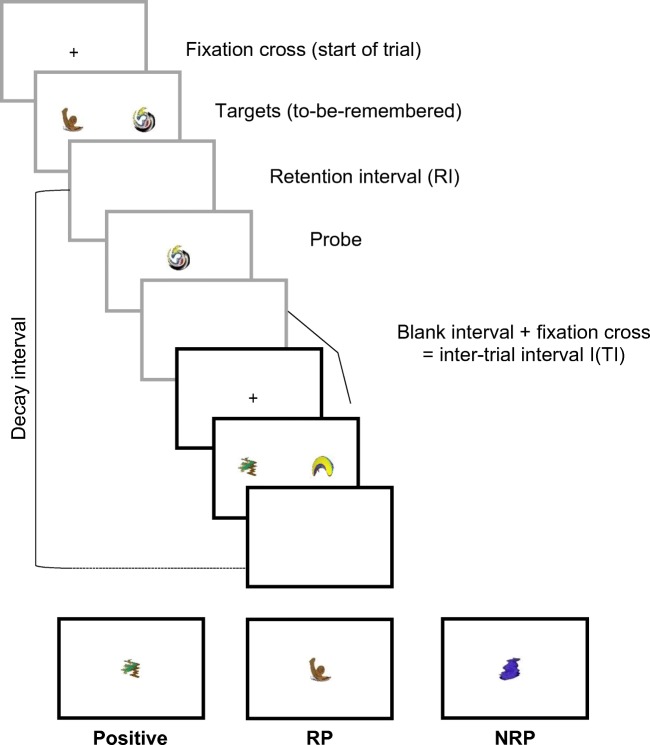
Schematic of the typical recent probes task in the reported experiments, using McKeown et al.’s (2014) stimuli. Here two trials are shown: trial *N* − 1 (gray boxes) and trial *N* (black boxes). Participants are requested to remember two target items over a delay, and then decide whether a single probe is a match for one of the targets. Nonmatch trials differ, with the probe either being novel (NRP) or a member of the target set from the previous trial (RP). Variables manipulated include the retention interval (RI) on a trial, the intertrial interval (ITI) and probe type

All five experiments used variants of the recent probes task (see Fig. 1). Participants reported normal or corrected-to-normal vision and were tested individually. The task was to remember two visual targets over a brief RI and decide whether a single probe matched one of the targets. The three standard probe types were employed, but the two mismatching probes—RP and NRP—were of primary interest, with both task accuracy (the proportion of correct responses) and response times being recorded. NRPs were either novel or had not been seen for multiple trials, whereas RPs matched a target from the previous trial (but never the probe). A ‘decay interval’ was computed by assessing the amount of time from offset of a target item on trial *N* − 1 to onset of the probe on trial *N*.

Two phenomena were of importance. Firstly, the effect of probe type provided a measurement of PI—less accurate or slower responding on RP than NRP trials would depict PI. Secondly, any reduction of PI would manifest as an ITI × Probe Type interaction, with improved performance on RP trials at longer ITIs. Such an interaction would provide evidence for a release from PI, in line with decay and temporal distinctiveness theories, and suggest that PI is time sensitive. Conversely, McKeown et al.’s (2014) active–passive conception predicts time-invariant PI and an absence of an interaction. Yet the active–passive theory also expects performance to decline over the RI, as actively maintained memories are subject to decay.

To assess these effects, repeated-measures ANOVAs were used, and violations to sphericity were corrected through the Greenhouse–Geisser adjustment. Null effects are theoretically relevant in this study, as an absence of an ITI × Probe Type interaction may reflect time-insensitive PI, and so Bayesian repeated-measures ANOVAs were also performed with JASP (JASP Team, 2018, Version 0.9.0.1; Wagenmakers et al., 2018). The analysis included both frequentist and Bayesian approaches.

For each main effect, a Bayes factor (BF_10_) was calculated, with values greater than 1 denoting support for the alternative hypothesis and values less than 1 denoting support for the null hypothesis (e.g., a BF_10_ of 5 would indicate that the data are five times more likely under the alternative rather than null hypothesis; a BF_10_ of 0.2 would indicate that the data are five times more likely under the null hypothesis). Interpretation of Bayes factors followed a recommendation from Jeffreys (1961; see also Dienes, 2014), where values exceeding 3 and 10 denote moderate and strong support for the alternative hypothesis, respectively (conversely, values less than 0.33 and 0.1 offer moderate and strong support for the null hypothesis, respectively). Bayes factors between 0.33 and 3 offer only limited or anecdotal support for either hypothesis, whereas values equaling 1 cannot differentiate the competing predictions. Here, such effects are considered inconclusive.

Assessment of interactions is more challenging, as the Bayesian analysis of complex experimental designs places greater emphasis on model comparison (for instance, comparing a model with an interaction against a model without an interaction). JASP offers a means of assessing the interaction based on Bayesian model averaging and the resulting BF_Inclusion_ gives a single value for each interaction, which is calculated by considering all models with a specific factor and comparing prior and posterior inclusion probability. When assessing interactions, the BF_Inclusion_ needs to be considered against models with the main effects alone, to see whether the interaction adds any value. The Bayesian analysis of interactions in this study uses BF_Inclusion_.

## Experiment 1

Experiment 1 tested McKeown et al.’s (2014) passive memory conception of a persisting PI by varying the ITI, leading to decay intervals of approximately 6 s to 21 s. The experiment also aimed to measure time-based forgetting of the actively maintained within-trial memory by varying the within-trial RI. To provide a more robust assessment of time-dependent forgetting, the positive trials were also included within the analysis.

### Method

#### Participants

Following McKeown et al. (2014), the intention was to recruit at least 15 participants for the experiment. The final sample included 18 psychology students from the University of Wolverhampton (16 females and two males) between the ages of18 and 38 years (*M* = 23.67 years, *SD* = 5.69 years).

#### Materials

Stimuli included 260 images developed by McKeown et al. (2014), which had originally been taken from Snodgrass and Vanderwart’s (1980) revised object databank (Rossion & Pourtois, 2004) and distorted into abstract and meaningless shapes (see Fig. 1). The experiment was run on a PC using E-Prime 2.0 software (Psychology Software Tools, Inc., www.pstnet.com/eprime). Stimuli were presented on an Iiyama ProLite P1905S 19-in. LCD monitor at a viewing distance of approximately 70 cm.

#### Design and procedure

The study matched the arrangements of McKeown et al. (2014, Experiment 1) using a within-groups design. Trials commenced with a fixation cross presented in the center of the screen for 500 ms, followed by two targets. Targets were displayed for 500 ms, and participants were instructed to remember both. A single probe stimulus was presented for up to 2 s after an unfilled RI lasting 1 s or 6 s. The task was to determine whether the probe matched either of the targets (using the “S” key for matches and the “L” key for nonmatches). After their response or after 2 s had elapsed, there was an unfilled interval lasting 500 ms or 5.5 s. The fixation cross was then displayed to indicate the beginning of the next trial, creating ITIs lasting 1 s or 6 s.

The probe matched one of the targets on 50% of trials and the remaining trials were equally distributed between RP and NRP trials. On RP trials, the probe matched a target seen on the *previous* trial, whereas on NRP trials the probe could not match any object seen for at least 48 trials. In addition, the combination of targets was unique. There were 16 practice trials and 192 experimental trials (96 positive, 48 RP and 48 NRP). Experimental trials were equally distributed between the four RI/ITI combinations and presented within four blocks of 48 trials. Participants received feedback following a block.

### Results

A 2 (ITI: 1 s vs. 6 s) × 2 (RI: 1 s vs. 6 s) × 3 (probe type: positive vs. RP vs. NRP) repeated-measures ANOVA assessed task accuracy (see Table 1). While overall accuracy was high, correct responding significantly decreased over the RI (1 s: *M* = 0.91; 6 s: *M* = 0.82), and these data offered extreme support for the alternative hypothesis, *F*(1, 17) = 28.69, *MSE* = 0.02, *p* < .001, η_p_^2^ = 0.63, BF^10^ = 2,555.98. The effect of ITI was also significant, ITI: *F*(1, 17) = 15.11, *MSE* = 0.003, *p* = .001, η_p_^2^ = 0.47, BF^10^ = 0.41, with accuracy modestly improving as the ITI was lengthened (1 s: *M* = 0.85; 6 s: *M* = 0.88). However, this effect was inconclusive and does not provide clear support for either hypothesis. Conversely, probe type was significant, and the corresponding Bayes factor indicated extreme support for the alternative hypothesis, *F*(2, 34) = 12.85, *MSE* = 0.07, *p* = .001, η_p_^2^ = 0.43, BF^10^ = 10,720,000,000. Šidàk post hoc tests revealed lower performance for positive probes (*M* = 0.77), in comparison to both NRP (*M* = 0.93, *p* = .001) and RP (*M* = 0.89, *p* = .028) trials. NRP accuracy was also significantly higher than RP accuracy (*p* = .035), highlighting PI.

**Table 1:** Mean proportion correct scores and response times in milliseconds (*SD*) for each probe and RI/ITI combination in Experiment 1

	Proportion correct			Response time		
RI/ITI	Positive	RP	NRP	Positive	RP	NRP
1 s / 1 s	0.83 (0.13)	0.91 (0.08)	0.95 (0.05)	772.03 (135.31)	854.45 (147.22)	812.26 (142.10)
1 s / 6 s	0.90 (0.13)	0.92 (0.10)	0.95 (0.07)	796.12 (135.58)	842.60 (134.10)	824.59 (145.72)
6 s / 1 s	0.64 (0.22)	0.86 (0.09)	0.91 (0.09)	872.31 (171.26)	871.88 (175.09)	828.61 (174.51)
6 s / 6 s	0.69 (0.24)	0.89 (0.11)	0.93 (0.10)	845.30 (162.26)	902.67 (173.90)	817.33 (159.30)

**Fig. 2:**
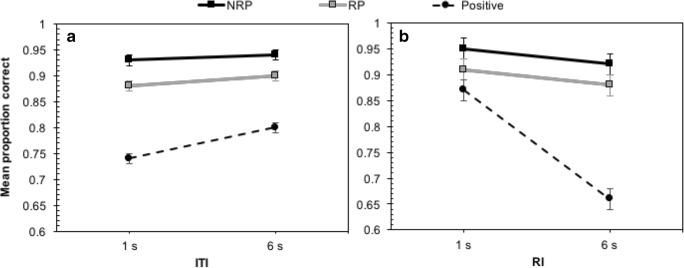
Proportion of correct responding in Experiment 1. Panel A shows the interaction between probe type and ITI, whereas Panel B shows the interaction between probe type and RI. Error bars show ±1 *SE* calculated for the factorial repeated measures design (Jarmasz & Hollands, 2009)

The interaction between probe type and ITI was conventionally significant, *F*(2, 34) = 3.33, *MSE* = 0.003, *p* = .048, η_p_^2^ = 0.16, BF_Inclusion_ = 0.15, with accuracy increasing at longer ITIs on positive trials, but not RP or NRP (see Fig. 2, Panel A). From the Bayesian perspective this interaction was unsupported, and there was no justification for including this interaction in the model. This discrepancy between the frequentist and Bayesian analysis is influenced by the Bayes factor used for interactions (BF_Inclusion_), which assesses whether the interaction adds value beyond the main effects alone. In this case, it did not, and the model was dominated by probe type. Indeed, at both ITIs, RP accuracy was around 4% lower than that recorded for NRP trials, showing persisting PI.

The Bayesian analysis consistently indicated minimal effect of the ITI, but there was stronger evidence for an RI × Probe Type interaction, as shown through both *p* and BF_Inclusion_, *F*(2, 34) = 19.21, *MSE* = 0.01, *p* < .001, η_p_^2^ = 0.43, BF_Inclusion_ = 6211.89 (see Fig. 2, Panel B). Holm–Šidàk-corrected *t* tests were used to assess this interaction, showing that accuracy for all probes declined as the RI increased from 1 s to 6 s. However, this was only significant on positive trials, which showed a performance drop of more than 20%, on average, NRP: *t*(17) = 1.53, *p* = .269, *d* = 0.44; RP: *t*(17) = 1.48, *p* = .157, *d* = 0.29; positive: *t*(17) = 6.65, *p* < .001, *d* = 2.32.

Next, response-time data were assessed with a 2 × 2 × 3 repeated-measures ANOVA (see Table 1). The analysis was only performed on trials featuring a correct response, and matching the accuracy data there was a significant effect of RI and strong support for the alternative hypothesis *F*(1, 17) = 7.29, *MSE* = 11462.69, *p* = .015, η_p_^2^ = 0.30, BF^10^ = 31.05. Reaction times slowed with increasing RI (1 s: *M* = 817.01 ms, 6 s: *M* = 856.35 ms). Probe type was also significant and strongly favored the alternative hypothesis, *F*(2, 34) = 4.84, *MSE* = 10875.98, *p* = .014, η_p_^2^ = 0.22, BF^10^ = 32.85. Responses to RP trials (*M* = 867.90 ms) were significantly slower than those on NRP trials (*M* = 820.70 ms, *p* = .005) and marginally slower than positive trials (*M* = 821.44 ms, *p* = .071), as confirmed through Šidàk post hoc tests.

The traditional ANOVA also showed one significant interaction between probe type and RI, *F*(2, 34) = 5.69, *MSE* = 3899.10, *p* = .007, η_p_^2^ = 0.25, which was influenced by an increase in response times at longer RIs on positive but not negative trials. However, responses on RP trials were slower than those on NRP trials at both 1 s and 6 s, and the Bayes factor was inconclusive (BF_Inclusion_ = 1.32). The model was instead dominated by the main effects of RI and probe type. There was minimal evidence for any other interactions and they were nonsignificant.

### Discussion

Experiment 1 documented persistent PI where performance was less accurate and slower on RP trials, in comparison to NRP trials, regardless of the ITI. These outcomes are broadly compatible with McKeown et al. (2014, Experiment 1). Strong time-based forgetting of actively maintained information was also observed, with accuracy declining and response times increasing at the longer RI. This effect was largely limited to positive trials, where participants were less successful at recognizing a match between the probe and one of the recently presented targets. The remaining experiments focus on this enduring PI as evidence for a passively maintained memory trace.

## Experiment 2

The manipulation of the RI in Experiment 1 meant that participants had to actively maintain representations over delays lasting up to 6 s. The effort involved in maintaining the target items may have strengthened the RP memory, heightening PI. In Experiment 2, a short, standardized RI was employed, reducing the time for active maintenance and permitting target items to be more rapidly forgotten once no longer relevant. This might alleviate PI, as less time is available to deploy active retention strategies during the RI. As in Experiment 1, the ITI was varied and if PI does decrease over time, a reduction in the recent probe effect should be observed at the longer ITIs.

### Method

#### Participants

The 22 participants (15 females and 7 males), between the ages of 18 and 48 years (*M* = 24.5 years, *SD* = 7.18 years), were either students or staff from the University of Wolverhampton.

#### Materials

The stimuli from Experiment 1 were used and the experiment was run on a PC using SuperLab 4.5 software (Cedrus Corporation, www.superlab.com). Stimuli were displayed on a HannsG HP191 19-in. LCD monitor at a viewing distance of approximately 80 cm.

#### Design and procedure

The experiment employed a fully within groups design and manipulated the probe type and ITI. The arrangements were broadly similar to Experiment 1, but the fixation cross remained on screen for 350 ms, the targets were presented for 750 ms and the RI was reduced to 350 ms. The probe was presented for a maximum of 2.5 s and participants were asked to press the “C” key to indicate a match and the “N” key to indicate a nonmatch. NRPs were novel. The probe was followed by a blank interval lasting 300 ms, 5 s or 10 s, creating ITIs of 650 ms, 5.35 s and 10.35 s. Participants completed nine practice trials and 144 experimental trials (72 positive, 36 RP and 36 NRP). The different probe types were equally distributed across the three ITIs, trials were presented within three blocks of 48 trials and block order was randomized for each participant. Blocks contained all three ITI durations and participants could take a break between blocks. No feedback was provided.

### Results and discussion

**Fig. 3:**
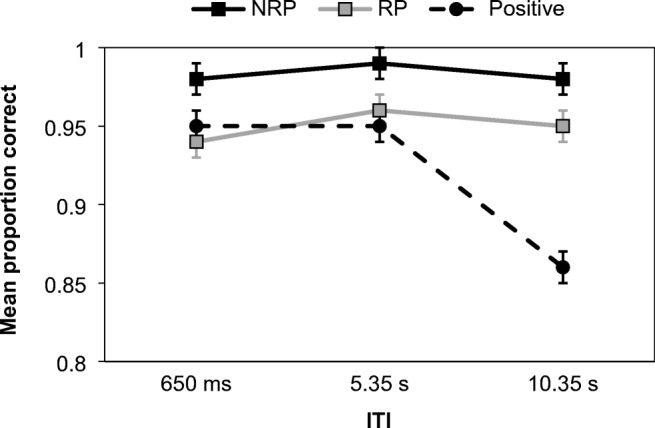
Mean proportion of correct responses for each probe type according to ITI in Experiment 2. Error bars show ±1 *SE*

This experiment was primarily concerned with the PI effect, which is revealed in the comparison between NRP and RP trials. However, as positive trials can provide information about how the task was approached, responses to matching probes were subjected to a separate analysis (due to experimenter error, only 16 of the 22 participants had positive trials available for analysis). As shown in Fig. 3, correct responding to positive trials was generally high, but declined at the longest ITI. A one-way repeated-measures ANOVA found a significant effect of ITI duration and extreme support for the alternative hypothesis, *F*(2, 30) = 32.37, *MSE* = 0.001, *p* < .001, η_p_^2^ = 0.68, BF^10^ = 308,269.05. Šidàk post hoc tests showed performance at the 10.35 s ITI to be poorer than both 650 ms (*p* < .001) and 5.35 s (*p* < .001). The latter two ITIs did not differ (*p* = .969). Analysis of response times on positive trials (see Table 2) also uncovered a significant effect, with strong support for the alternative hypothesis, *F*(2, 30) = 15.01, *MSE* = 2554.19, *p* < .001, η_p_^2^ = .50, BF^10^ = 39.48. Šidàk post hoc tests found quicker responding at the 650 ms ITI in comparison to both 5.35 s (*p* = .005) and 10.35 s (*p* = .001). The latter two ITIs did not differ (*p* = .303). Thus, outcomes on positive trials in both frequentist and Bayesian approaches were consistent.

**Table 2:** Mean response times in milliseconds (*SD*) according to probe type and ITI in Experiment 2

ITI	Positive	RP	NRP
650 ms	711.00 (112.33)	796.32 (172.67)	770.50 (199.29)
5.35 s	781.20 (143.97)	787.55 (178.51)	755.13 (138.75)
10.35 s	805.22 (164.10)	804.71 (194.59)	777.00 (173.78)

*Note.* Responses to positive trials were based on 16 participants, whereas RP and NRP trials came from 22 participants

To assess PI, the proportion of correct responding to negative probes (see Fig. 3) was assessed using a 2 (probe type: RP vs. NRP) × 3 (ITI: 650ms vs. 5.35 s vs. 10.35 s) repeated-measures ANOVA, which found a significant main effect of probe type, *F*(1, 21) = 9.62, *MSE* = 0.004, *p* = .005, η_p_^2^ = 0.31, BF^10^ = 87.78. In addition to the significant result, there was very strong evidence for the alternative hypothesis, resulting from poorer performance on RP (*M* = 0.95) than NRP (*M* = 0.98) trials. Crucially, there was no significant effect of ITI, *F*(2, 42) = 0.65, *MSE* = 0.003, *p* = .528, η_p_^2^ = 0.03, BF^10^ = 0.13, and the data were 7.94 times more likely under the null than alternative hypothesis. There was also no interaction, *F*(2, 42) = 0.83, *MSE* = 0.002, *p* = .442, η_p_^2^ = 0.04, BF_Inclusion_ = 0.11, with the model being dominated by the effect of probe type alone.

Another 2 × 3 ANOVA then assessed the response time data (see Table 2). The effect of probe type was significant, *F*(1, 21) = 4.91, *MSE* = 5513.61, *p* = .038, η_p_^2^ = 0.19, BF^10^ = 1.28, with slower responding to RP (*M* = 796.19 ms) than NRP trials (*M* = 767.55 ms), but these data did not offer convincing support for either hypothesis and were inconclusive in the Bayesian analysis. Both the ITI effect and the interaction were again nonsignificant (*F*s < 0.6, *p*s > .5), which was supported by the Bayesian analysis (ITI: BF^10^ = 0.12; interaction: BF_Inclusion_ = 0.13). In summary, the present experiment replicated the recent probe effect of Experiment 1, but primarily for task accuracy, and this PI effect did not seem to diminish over time.

## Experiment 3

The time-invariant PI observed in the prior two experiments is notable given the lengthy decay intervals employed (Experiment 1: approx. 6–21 s; Experiment 2: 5–15 s). This effect may indicate that passively maintained memories do not decay, but to more severely test this notion, Experiment 3 extended the ITI even further—up to 32 s—creating a decay interval of 39 s (the other ITI, 8 s, led to a decay interval of 15 s). Experiment 3 (and the subsequent two experiments) also increased the sample size, to improve statistical power and increase the likelihood of detecting a reduction in PI. A previous study detecting a reduction in PI over time (Mercer & Duffy, 2015) employed a sample of 29 individuals, and effort was made to obtain a similar sample size in the final three experiments.

### Method

#### Participants

Thirty undergraduate psychology students (29 females and one male) from the University of Leeds (mean age = 20.73 years, *SD* = 4.23 years) completed the experiment.

#### Materials

Stimuli matched Experiment 1 and the study was run on a PC using E-Prime 2.0 software. The stimuli were presented on a Dell 1708FP monitor at a viewing distance of approximately 70 cm.

#### Design and procedure

The experiment used a fully within groups design and manipulated the probe type and ITI. Each trial began with a central fixation cross lasting 2 s followed by the two target stimuli, displayed for 500 ms. After a 2-s RI, the probe was displayed for up to 2 s. Participants responded “1” to indicate a match and “3” to indicate a nonmatch. The next trial began after a delay of 6 s or 30 s, creating ITIs lasting 8 s or 32 s (interval + fixation cross). Participants completed 16 practice trials and 96 experimental trials (48 positive, 24 RP and 24 NRP). NRPs were novel. Experimental trials were equally distributed across the conditions and presented within four blocks of 24 trials (12 positive, 6 RP and 6 NRP). Experimental conditions were randomized within blocks.

### Results and discussion

**Fig. 4:**
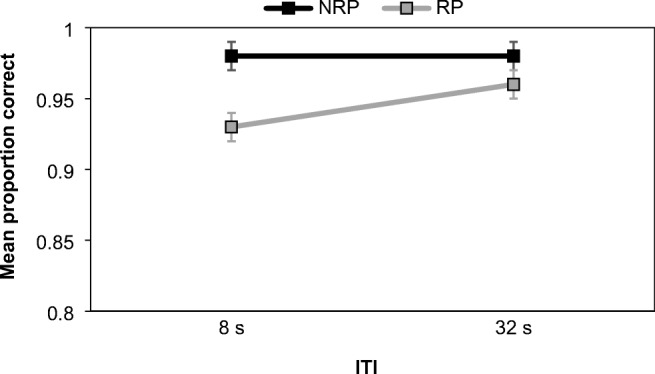
Mean proportion of correct responses on RP and NRP trials according to ITI in Experiment 3. Error bars show ±1 *SE*

Responses to positive trials were not retained in Experiment 3 and so the analysis only focused on the negative probes. Data were examined using a 2 (probe type: RP vs. NRP) × 2 (ITI: 8 s vs. 32 s) repeated-measures ANOVA. For task accuracy (see Fig. 4), both main effects were significant, but convincing evidence for the alternative hypothesis was only recorded for the probe type. Accuracy for RP trials (*M* = 0.94) was lower than NRP trials (*M* = 0.98), *F*(1, 29) = 13.63, *MSE* = 0.003, *p* = .001, η_p_^2^ = 0.32, BF^10^ = 1349.97, and this outcome provided extreme support for the alternative hypothesis, demonstrating PI. The ITI effect was driven by a very modest increase in performance at the 32 s ITI (*M* = 0.97), in comparison to that at 8 s (*M* = 0.96), *F*(1, 29) = 4.44, *MSE* = 0.001, *p* = .044, η_p_^2^ = 0.13, BF^10^ = 0.57, but this effect was inconclusive.

The interaction was also significant, *F*(1, 29) = 5.64, *MSE* = 0.002, *p* = .024, η_p_^2^ = 0.16, BF_Inclusion_ = 1.51, and there was an improvement in accuracy on RP trials as the ITI was lengthened, highlighting a release from PI. However, there was no convincing support for the interaction from the Bayesian perspective, showing a discrepancy with the frequentist analysis. As noted above, the BF_Inclusion_ score assesses the value of retaining the interaction within the model, by comparing it against models based on main effects alone. In this case, the model was dominated by the effect of probe type and RP performance remained lower than NRP trials at both ITIs.

A similar two-way repeated-measures ANOVA assessing the reaction time data yielded no main effects, no interactions and evidence more congruent with the null hypothesis. In summary, Experiment 3 found a robust recent probe effect for task accuracy. While PI was modestly alleviated after a 32 s ITI, the Bayesian analysis suggested little justification for including the interaction within the model, supporting the notion of enduring PI.

## Experiment 4

Experiments 1–3 found that old visual representations persist and disrupt current task performance over lengthy intervals. Yet in some circumstances it would be helpful to more effectively manage and regulate PI. The present experiment tested this idea, being influenced by recent evidence suggesting that individuals can control forgetting. For example, Festini and Reuter-Lorenz (2014) combined the recent probes task with a directed forgetting procedure and found that PI for verbal stimuli was prevented when participants were instructed to forget one part of the target array after encoding. Williams, Hong, Kang, Carlisle, and Woodman (2013) reported a similar effect.

Retrospectively cueing an object during the RI also has positive effects on subsequent retention (e.g., Griffin & Nobre, 2003; Landman, Spekreijse, & Lamme, 2003). This ‘retro-cueing’ effect can be explained in a number of ways (see Souza & Oberauer, 2016), but one account states that the cued item is protected from decay, whereas uncued items are susceptible to time-based forgetting. The alternative ‘removal hypothesis’ states that uncued items are marked as irrelevant and then actively removed from the memory buffer (Souza & Oberauer, 2016).

The role of time in the retro-cueing effect was demonstrated by Pertzov, Bays, Joseph, and Husain (2013), who had participants remember simple visual stimuli over RIs of different durations. Including a valid retro-cue was beneficial and preserved the object over the RI, whereas uncued and invalidly cued items were subject to temporal forgetting. Pertzov et al. argued that validly cued objects are held in a privileged state, but this has a cost for uncued items, which are forgotten. Nevertheless, other studies report uncued objects do persist in memory (e.g., Gressmann & Janczyk, 2016; Schneider, Mertes, & Wascher, 2015; van Moorselaar, Olivers, Theeuwes, Lamme, & Sligte, 2015).

Experiment 4 incorporated a retro-cue into the recent probes task on half of the trials. Arrangements were similar to Experiments 1–3 except one condition featured a cue during the RI (the “CP” or “cue present” condition) denoting the target to be remembered, and so when the probe occurred, participants had to determine whether it matched the cued object. On CP-positive trials, the probe did match the cued object; on CP-NRP trials, there was not a match, and the probe was novel; but on CP-RP trials the probe matched the *uncued* target from the previous trial. So, CP-RP trials included a cue, but the probe itself had not been cued when displayed as a target. In the “CA” or “cue absent” condition, the cue was removed and both targets had to be remembered (and again there were three probe types: CA-Positive, CA-NRP, and CA-RP). The ITI was 800 ms or 8.3 s, creating decay intervals of 8.3 s and 15.3 s, respectively.

If participants can forget uncued items, CP-RP stimuli should produce less PI and suffer from time-based decay, whereas this should not occur in the CA-RP condition. In contrast, the active–passive conception predicts an enduring PI effect, which should not be eliminated by the presence of a retro-cue.

### Method

#### Participants

The final sample included 31 students from the University of Wolverhampton (26 females and five males) between the ages of 18 and 47 years (*M* = 24.81 years, *SD* = 8.38 years). As in Experiments 1 and 3, participants had 2 s to respond, but some struggled with this. Individuals with 10%+ missing data were excluded.

#### Materials

**Fig. 5:**
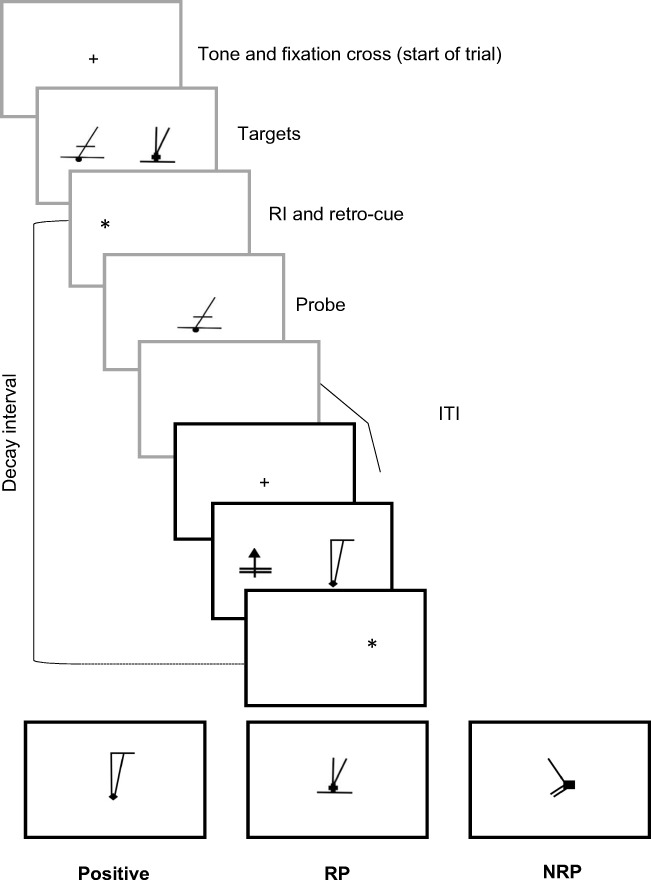
Diagram depicting two trials in Experiment 4: trial *N* − 1 (gray boxes) and trial *N* (black boxes). A postencoding retro-cue was presented on half of the trials, for 100 ms, and indicated the target that should be remembered. On the remaining half of trials, the retro-cue was removed and both targets had to be remembered. The three standard probe types were employed, but when a retro-cue was presented the RP item did not need to be remembered and could be discarded from memory

This experiment involved numerous trials needing unique stimuli. To achieve this, a new set of visual objects were created. Each stimulus contained three lines of varying lengths and orientations along with a single shape (a circle, a square, a triangle, a diamond, a star, a cross, an “L” and an “X”). Each shape was used in the construction of 75 stimuli, creating 600 images, all of which were black and presented against a white background (see Fig. 5). In total, 576 of these stimuli were used on experimental trials (and 10 on practice trials). Images were randomly paired to form the targets.

Other stimuli included a pure tone warning signal (4.8 kHz) presented at approximately 65 dB and generated using Audacity (Version 2.0.3), and a black asterisk that served as the retro-cue (Calibri type size: 96). The cue was presented in the same location as the left or right target. The experiment was run on a PC using SuperLab 5 software and a Lenovo ThinkVision 24-in. LCD monitor from a viewing distance of approximately 70 cm.

#### Design and procedure

A within groups design was used, with the presence of the cue, the ITI and the probe type being manipulated (see Fig. 5). Each trial commenced with a tone (lasting 300 ms) and a central fixation cross (lasting 100 ms and presented 200 ms after the tone onset). Targets were displayed for 500ms and followed by a 2.5-s RI. On CA trials the delay was unfilled, but on CP trials the cue was presented for 100ms in the position of one of the targets. The cue was shown 550 ms after the offset of the targets and the left and right targets were cued an equal number of times. The probe was shown for a maximum of 2 s and the next trial began after an interval of 500 ms or 8 s, creating ITIs of 800 ms and 8.3 s.

On CP trials, participants judged whether the probe matched the cued target (pressing “M” for match and “Z” for nonmatch). When a cue was present, the non-cued target would never be shown as a probe on that trial. This was intended to encourage participants to focus exclusively on the cued object (and cue validity may be important; Gunseli, van Moorselaar, Meeter, & Olivers, 2015). On CA trials, participants had to determine whether the probe matched either target. Once again, the probe could take three forms (positive, RP, and NRP), and NRP stimuli were novel. On a CA trial, the RP item could be either of the targets seen on the previous trial, whereas on CP trials the RP stimulus was always the object that was not cued.

Participants completed four practice trials (two with a cue and two without) and 256 experimental trials (64 trials for each cue/ITI combination, including 32 positive trials, 16 RP trials and 16 NRP trials). The trials were organized into four blocks (two CA and two CP) that contained both ITIs and all probe types. The trial arrangement within a block was fixed, but the order of blocks was randomly determined. A break was available after two blocks. No feedback was provided.

### Results and discussion

Trials on which the participant did not respond within 2 s or pressed an invalid button (neither “M” or “Z”) were excluded (fewer than 2.5% of trials, on average).

Firstly, responding on positive trials was examined (see Table 3) and accuracy was assessed using a 2 (cue: CP vs. CA) × 2 (ITI: 800 ms vs. 8.3 s) repeated-measures ANOVA. There was a significant effect of the cue and extreme support for the alternative hypothesis, *F*(1, 30) = 14.93, *MSE* = 0.01, *p* = .001, η_p_^2^ = 0.33, BF^10^ = 446.05, with higher accuracy when the cue was present (*M* = 0.83) than absent (*M* = 0.75). The effect of ITI was also significant and offered support for the alternative hypothesis, *F*(1, 30) = 12.54, *MSE* = 0.01, *p* = .001, η_p_^2^ = 0.30, BF^10^ = 17.67. There was an improvement in accuracy as the ITI was extended (800 ms: *M* = 0.76, 8.3 s: *M* = 0.82). However, the interaction was nonsignificant and did not contribute to the model beyond the two main effects, *F*(1, 30) = 2.75, *MSE* = 0.01, *p* = .108, η_p_^2^ = 0.08, BF_Inclusion_ = 0.47.

**Table 3:** Mean proportion correct scores and response times in milliseconds (*SD*) on positive trials in Experiment 4

	Proportion correct		Response time	
	800 ms ITI	8.3 s ITI	800 ms ITI	8.3 s ITI
CP-positive	0.81 (0.13)	0.85 (0.14)	697.97 (147.43)	727.41 (131.39)
CA-positive	0.71 (0.13)	0.79 (0.13)	808.00 (135.09)	819.98 (127.28)

Response times were assessed in the same manner. Another significant effect of cue type was revealed and there was extreme support for the alternative hypothesis *F*(1, 30) = 45.06, *MSE* = 7059.10, *p* < .001, η_p_^2^ = 0.60, BF^10^ > 1,000,000. Responses were faster with a cue (*M* = 712.69) than without (*M* = 813.99). The effect of ITI was significant, *F*(1, 30) = 4.86, *MSE* = 2736.09, *p* = .035, η_p_^2^ = 0.14, BF^10^ = 0.43, and responses were slightly quicker after the shorter ITI (800 ms: *M* = 752.98; 8.3 s: *M* = 773.69). However, the Bayesian analysis was inconclusive. The interaction was nonsignificant and there was little justification for including this interaction in the model, *F*(1, 30) = 1.28, *MSE* = 1839.88, *p* = .266, η_p_^2^ = 0.04, BF_Inclusion_ = 0.36.

**Fig. 6:**
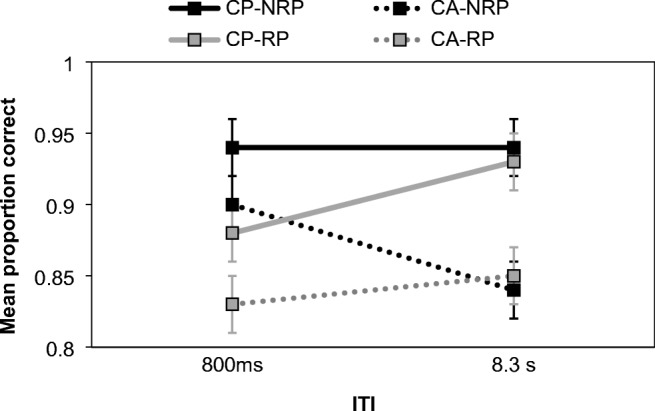
Mean proportion of correct responses in Experiment 4. Data show RP and NRP conditions for each ITI, according to whether a retro-cue was present (CP) or absent (CA). Error bars show ±1 *SE*

PI was assessed using a 2 (probe type: RP vs. NRP) × 2 (cue: CP vs. CA) × 2 (ITI: 800 ms vs. 8.3 s) repeated-measures ANOVA (see Fig. 6). There was a significant effect of probe type, *F*(1, 30) = 14.49, *MSE* = 0.004, *p* = .001, η_p_^2^ = 0.33, BF^10^ = 4.44, denoting moderate support for the alternative hypothesis and replicating the previous results—accuracy was lower on RP (*M* = 0.87) than NRP (*M* = 0.90) trials. Performance was also better when a cue was present (*M* = 0.92) than when it was absent (*M* = 0.86), *F*(1, 30) = 26.26, *MSE* = 0.01, *p* < .001, η_p_^2^ = .47, BF^10^ = 1,689,000, with the effect being significant and offering extreme support for the alternative hypothesis. The main effect of ITI was not significant, *F*(1, 30) = 0.43, *MSE* = 0.01, *p* = .518, η_p_^2^ = 0.01, BF^10^ = 0.16, and the Bayesian analysis showed that these data were 6.17 times more likely under the null hypothesis.

The traditional ANOVA found an interaction between ITI and cue, *F*(1, 30) = 5.81, *MSE* = 0.01, *p* = .022, η_p_^2^ = 0.16, BF_Inclusion_ = 1.23, and ITI and probe type, *F*(1, 30) = 8.89, *MSE* = 0.01, *p* = .006, η_p_^2^ = 0.23, BF_Inclusion_ = 25.29. The former interaction was driven by improved accuracy at the longer ITI for CP, but not CA trials; however, the Bayesian analysis suggested limited justification for including this interaction within the model. Much better support was provided for the ITI and probe type interaction, which is shown in Table 4. A simple effects analysis revealed an improvement in accuracy on RP trials as the ITI was lengthened, *F*(1, 30) = 7.22, *p* = .012, but no differences between the two ITIs on NRP trials, *F*(1, 30) = 2.74, *p* = .108. Of particular relevance was the three-way interaction, as this would determine whether any time-based recovery from PI was particularly likely on cued trials. This was unsupported and non-significant, *F*(1, 30) = 5.81, *MSE* = 0.01, *p* = .48, η_p_^2^ = 0.02, BF_Inclusion_ = 0.31. Additionally, the probe type x cue interaction was nonsignificant and there was no evidence for retaining it based on the Bayes factor, *F*(1, 30) = 0.03, *MSE* = 0.004, *p* = .872, η_p_^2^ = 0.16, BF_Inclusion_ = 0.20.

**Table 4:** Mean proportion correct responses (*SD*) for RP and NRP probes according to ITI in Experiment 4

ITI	RP	NRP
800 ms	0.85 (0.12)	0.92 (0.08)
8.3 s	0.89 (0.09)	0.89 (0.12)

Another 2 × 2 × 2 repeated-measures ANOVA assessing response times found just one significant main effect. Participants were significantly faster to respond on CP (*M* = 758.25 ms) than CA (*M* = 852.48 ms) trials, *F*(1, 30) = 41.68, *MSE* = 13,206.34, *p* < .001, η_p_^2^ = 0.58, BF^10^ > 1,000,000. This effect showed extreme support for the alternative hypothesis, but all other results were nonsignificant and compatible with the null hypothesis.

Experiment 4 found the recent probe effect for accuracy data, highlighting PI, and the presence of a retro-cue was beneficial, leading to faster and more accurate responding on both positive and negative trials, in line with past work (see Souza & Oberauer, 2016). Unlike Experiments 1–3, PI declined slightly over a longer ITI, but this did not seem to be reliably affected by the cue, with performance improving over the ITI on RP trials for both CP and CA conditions. Thus, the present results suggest that there is PI even when a cue offers a reliable instruction for the interfering item to be discarded, but PI did modestly diminish as time passed (although the ITI × Probe Type interaction was partly influenced by the NRP condition, where performance unexpectedly declined at the longer ITI in the CA condition). The last experiment attempted to replicate this interaction and further investigate the role of the retro-cue in reducing PI.

## Experiment 5

Experiment 4 tested whether PI could be alleviated when a retro-cue instructed participants to forget the RP item. This idea was unsupported, but the RP stimulus itself was never cued. This allowed a distinction to be made between conditions in which the RP stimulus either had to be maintained over the RI (CA) or did not (CP). Yet a limitation with this design was that the RP stimulus had to be retained alongside the target in the CA condition, and so could not be exclusively prioritized.

In this final experiment, a retro-cue was presented on all trials, and participants were instructed to only remember the cued item and determine whether it matched the probe. The key manipulation concerned the type of RP stimulus. On uncued RP trials, the RP stimulus had not been cued when presented on the previous trial and therefore should not have been maintained over the RI, whereas on cued RP trials this stimulus had been cued (but not presented as the current probe). In this latter arrangement, the RP stimulus may be more enduring and exert a stronger interfering effect. Following previous experiments, the ITI was varied, and this experiment served two purposes: (1) it provided a more direct test of the role of active maintenance in PI; (2) it offered an attempt to replicate the ITI × Probe Type interaction reported in Experiment 4.

### Method

#### Participants

The final sample included 25 (predominantly female) students from the University of Wolverhampton. As in Experiment 4, some participants struggled to respond within the 2-s window (or consistently pressed invalid buttons) and were excluded if 10%+ trials were affected. This applied to five participants.

#### Materials

Stimuli and equipment were identical to those of Experiment 4.

#### Design and procedure

The study was a within groups design and manipulated the ITI (800 ms or 8.3 s) and probe type (positive, NRP, cued RP, and uncued RP). The procedure matched Experiment 4, except the retro-cue was used on every trial. This allowed two different RP trials to be created. On cued RP trials, the RP stimulus was presented as a target on trial *N* − 1 and subsequently cued during the RI. However, it was not presented as a probe until trial *N*. Uncued RP trials were similar, except the RP stimulus was not cued on trial *N −* 1 – this matched the arrangement for the CP-RP trials in Experiment 4. Participants were asked to determine whether the probe matched the cued target on that trial.

There were 192 experimental trials, with 96 trials for each ITI (48 positive, 16 NRP, 16 cued RP and 16 uncued RP). Trials were arranged into four blocks that followed a predetermined pattern, but the block order was random.

### Results and discussion

Trials on which the participant did not respond within 2 s or pressed an invalid button were excluded (fewer than 2% of trials, on average). The first analysis examined responding on positive trials, comparing the short and long ITIs using traditional and Bayesian paired-samples *t* test (with a two-tailed hypothesis). For task accuracy, the proportion of correct responses was slightly lower when the ITI was 800 ms (*M* = 0.78) than 8.3 s (*M* = 0.82). This was conventionally significant, but inconclusive from the Bayesian perspective, *t*(24) = −2.41, *p* = .024, *d* = -0.49, BF^10^ = 2.30. For response times, participants were quicker at responding on trials with the short (*M* = 721.93 ms) than long (*M* = 738.95) ITI, *t*(24) = −1.24, *p* = .226, *d* = 0.25, BF^10^ = 0.42. This effect was nonsignificant, but inconclusive.

**Fig. 7:**
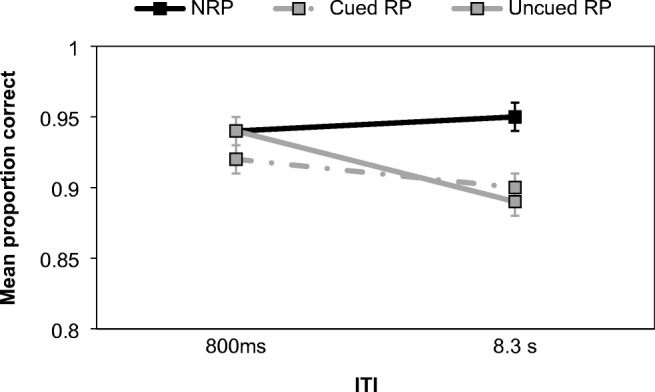
Mean proportion of correct responses on NRP, cued RP cued and uncued RP trials according to ITI in Experiment 5. Error bars show ±1 *SE*

PI was then assessed using a 2 (ITI: 800 ms vs. 8.3 s) × 3 (probe type: NRP vs. cued RP vs. uncued RP) repeated-measures ANOVA on task accuracy (see Fig. 7). There was a significant main effect of probe type, *F*(2, 48) = 7.63, *MSE* = 0.003, *p* < .001, η_p_^2^ = 0.24, BF^10^ = 3.36, and moderate support for the alternative hypothesis. Šidàk post hoc tests showed that performance on NRP trials (*M* = 0.95) exceeded cued RP (*M* = 0.91, *p* = .007) and uncued RP (*M* = 0.92, *p* = .016) trials, but the latter two conditions did not differ (*p* = .934). The effect of ITI was nonsignificant but inconclusive from the Bayesian perspective, *F*(1, 24) = 3.48, *MSE* = 0.004, *p* = .075, η_p_^2^ = 0.13, BF^10^ = 0.66. The interaction was also nonsignificant, *F*(2, 48) = 2.43, *MSE* = 0.01, *p* = .099, η_p_^2^ = 0.09, BF_Inclusion_ = 1.55, and inconclusive based on the Bayes factor. Importantly, this result was not in line with Experiment 4. As seen in Fig. 7, accuracy *decreased* at the longer ITI for both RP trial types, while remaining constant for NRP stimuli.

Another two-way ANOVA assessing response times yielded only one reliable effect. Participants were slower to respond at the long (*M* = 801.35 ms) than the short (*M* = 749.95 ms) ITI, *F*(1, 24) = 26.34, *MSE* = 3762.28, *p* < .001, η_p_^2^ = 0.53, BF^10^ = 115,247.91, and there was extreme support for the alternative hypothesis. Conversely, the main effect of probe type, *F*(2, 48) = 0.74, *MSE* = 3550.89, *p* = .482, η_p_^2^ = 0.03, BF^10^ = 0.10, and the interaction, *F*(2, 48) = 0.46, *MSE* = 3180.70, *p* = .633, η_p_^2^ = 0.02, BF_Inclusion_ = 0.16, were nonsignificant and unsupported by the Bayesian analysis.

In summary, PI was present for accuracy data, but both types of RP stimuli damaged performance. Thus, whether the RP item had been cued (and actively maintained) or not cued (and discarded) did not affect PI. This experiment also found no support for a reduction in PI over time.

## General discussion

The expression of PI was exploited in the present study to explore the persistence of old visual memories over time. Given demonstrations of rapid time-dependent forgetting in visual WM (e.g., Ricker & Cowan, 2010, 2014), it is reasonable to expect PI to vanish over longer intervals, and such an effect is predicted by decay and temporal distinctiveness theories. Conversely, McKeown et al.’s (2014) active–passive conception expects time-insensitive PI. The present data were more compatible with this form of enduring passive memory trace.

PI was manifested as a reduction in accurate responding on RP in comparison to NRP trials across all five experiments. This was found with both the frequentist and Bayesian analyses. Response times to RP stimuli were also slowed in Experiment 1, though generally the PI effect was confined to accuracy. Our demonstration of PI is compatible with previous studies (e.g., Cyr et al., 2017; Hartshorne, 2008; Makovski & Jiang, 2008; McKeown et al., 2014; Mercer & Duffy, 2015) and highlights its role in short-term forgetting. More significantly, the PI effect was largely robust over time, as assessed through an interaction between probe type and ITI length. In Experiments 2 and 5, this interaction was nonsignificant, and there was no justification for retaining it from the Bayesian perspective. In Experiment 1, the interaction was significant, but performance only changed over the ITI on positive trials—the disadvantage on RP compared with NRP trials endured over time. Furthermore, there was no support for retaining that interaction on the basis of the Bayes factor.

Somewhat better evidence for time-sensitive PI was found in Experiments 3 and 4. Experiment 3 used a very long ITI, allowing ample time for old memories to be forgotten, and the frequentist analysis suggested a modest recovery in performance at the longest ITI. Yet the Bayesian analysis was inconclusive and accuracy on RP trials was still lower than NRP after the 32-s ITI. The only case where both frequentist and Bayesian analyses supported the ITI × Probe Type interaction was Experiment 4. Here, accuracy on RP trials improved as the ITI was lengthened, indicating a recovery from PI over a longer delay. Yet this interaction was partly influenced by an unexpected decrease in the CA-NRP condition at the longer ITI, and it could not be replicated in Experiment 5. In summary, the combined evidence was consistent with robust and largely time-invariant PI.

Such PI appears different to the more limited, immediate effects reported in some prior studies (e.g., Makovski & Jiang, 2008), although the present results are congruent with Berman et al.’s (2009) investigation of PI, which estimated that a delay of 78 s would be required to eliminate the RP–NRP difference (for response times). Of course, the current study used abstract and unfamiliar visual stimuli that are likely to be harder to maintain through intentional maintenance strategies such as rehearsal.

Yet the present experiments uncover time-based effects that appear paradoxical: old and redundant items from previous trials persist over lengthy intervals, yet intentionally maintaining a single item over a short delay is difficult (note the drop in performance in Experiment 1 as the RI was extended). It should be noted that the two time-based effects are manifested through different responses: on positive trials, where there is time-dependent forgetting, the participant must determine whether there is an identical match between a probe and one of the targets. On negative trials, the participant must only reject the probe, and generally this is successfully accomplished. Table 5 shows the mean proportion of correct responding (and standard deviations) in the present experiments according to probe type. In all cases, responding to positive trials was less accurate than responding to negative trials, and this became more noticeable at longer RIs. The memory requirements on positive and negative trials may differ—only in the former case is a precise representation required to make a correct response. Furthermore, forgetting over the RI was greatly reduced on RP and NRP trials, in comparison to positive trials, as shown in Experiment 1 and further revealed in Table 5.

**Table 5:** Mean proportion correct responses (*SD*) according to probe type in Experiments 1–5

Experiment	RI length	Positive	RP	NRP
1 (short RI)	1 s	0.87 (0.13)	0.91 (0.07)	0.95 (0.04)
1 (long RI)	6 s	0.66 (0.23)	0.88 (0.09)	0.92 (0.08)
2	0.35 s	0.92 (0.06)	0.95 (0.05)	0.98 (0.02)
3	2 s	–	0.94 (0.06)	0.98 (0.03)
4	2.5 s	0.79 (0.11)	0.87 (0.10)	0.90 (0.09)
5	2.5 s	0.80 (0.09)	0.91 (0.11)	0.95 (0.10)

The notion of rapid forgetting of actively maintained information and slower loss of residual representations of the McKeown et al. (2014) model is consistent with the work of Logie and colleagues. In their first experiment Shimi and Logie (2019) used a change detection task, with participants remembering arrays of four or six objects. The array was repeated throughout the experiment, and this was beneficial—arrays of six items repeated multiple times were learnt, particularly within the first 40 trials. Their second experiment explored memory for six-item arrays only, but memory was tested using a visual reconstruction procedure. Clear evidence for learning was again demonstrated, especially within the first ~20 trials. These findings suggest that some visual information from a trial must persist and Shimi and Logie made a distinction between a short-lived memory that is highly vulnerable to interference from subsequent input, and a weaker, residual trace generated across trials. These ideas, emerging from a different paradigm, are compatible with the active–passive conception, and highlight the need to consider residual representations that do not neatly fit the description of a traditional short-term or long-term memory. Logie, Brockmole, and Vandenbroucke (2009) suggested that although visual short-term memory may be fragile, nevertheless feature bindings established through short-term memory can influence long-term learning. For such learning to happen, some information must survive beyond a trial. Interestingly, a robust PI for visual memoranda was recently reported in rhesus monkeys by Devkar and Wright (2016) over decay intervals between about 19 s and 58 s.

Future research would benefit from exploring these issues in more depth, particularly by interrogating the nature of passively held residual memories. The trace could be viewed as a lingering WM, reflecting the remaining contents of the immediately preceding trial, or it could be an LTM. Time-dependent forgetting over the RI may reflect a decaying WM, whereas PI is driven by a more robust LTM. While appealing, there are some reasons to doubt this interpretation. Individual target stimuli were unfamiliar to participants and briefly presented as a target once. Although this does not eliminate the possibility that new LTMs were rapidly formed, it is unclear why such memories could not be used to prevent time-dependent forgetting over the RI. Alternatively, the PI effect may be better interpreted as a decision-making phenomenon that occurs during retrieval. Oberauer, Awh, and Sutterer (2017) proposed that responses to the probe involve a competition between a familiarity signal from LTM and the available content of WM. Familiarity with the probe can be used to make a decision, which is beneficial on positive trials but detrimental on RP trials. Specifically, familiarity with the RP will lead to an incorrect decision. More direct experimentation capable of distinguishing these accounts will help better comprehend the nature of PI for visual stimuli.

So, we conclude with this fundamental puzzle: A target that appears to decay rather rapidly within trials is nevertheless producing PI on future trials. The active–passive conception advanced by McKeown et al. (2014) addresses this puzzle. Here, it is proposed the attention-based maintenance that refreshes or reactivates the immediate memory trace actually has the parallel negative effect of introducing noise into the representation. This might occur if the neural bases of the trace were entered into, or exchanged between, a WM buffer for prioritized attention and a residual or passive store; the assumption in the model is that such translation is never perfect. When the trace is within prioritized focal attention it is available to guide recall responses (see, for example, Ricker & Cowan, 2010); when in the residual or passive form, it is not. Thus, items held within the passive store have a more enduring time course *precisely because* they escape the translation involved in bringing a recent memory trace into the focus of attention, following the termination of the trial on which that item occurred (i.e., throughout the subsequent ITI). In conclusion, there is a passive form of memory trace in visual memory that remains stable over time and is difficult to control. Conversely, actively maintained representations are subject to rapid forgetting.
